# Incidence of new-onset diabetes with 1 mg versus 4 mg pitavastatin in patients at high risk of developing diabetes during a 3-year follow-up

**DOI:** 10.1186/s12933-019-0969-z

**Published:** 2019-11-21

**Authors:** Han Saem Jeong, Soon Jun Hong, Serhim Son, Hyonggin An, Hyungdon Kook, Hyung Joon Joo, Jae Hyoung Park, Cheol Woong Yu, Do-Sun Lim

**Affiliations:** 1 Heart Diseases Research Institute, Dr. Jeong’s Heart Clinic, Jeonju, Republic of Korea; 2 Department of Cardiology, Cardiovascular Center, Korea University Anam Hospital, 126-1, 5ka, Anam-dong, Sungbuk-ku, Seoul, 136-705 Republic of Korea; 3 Department of Biostatistics, Korea University, Seoul, Republic of Korea

**Keywords:** Acute coronary syndrome, New-onset diabetes, Metabolic syndrome, Pitavastatin

## Abstract

**Background:**

Statin therapy reduces the risk of cardiovascular events across a broad spectrum of patients; however, it increases the risk of new-onset diabetes (NOD). Although the highest dose pitavastatin is considered to not be associated with NOD, there are limited data regarding the impact of long-term highest dose pitavastatin use on the development of NOD in patients at high risk of developing diabetes. Therefore, we prospectively compared the differences in the development of NOD between the lowest and the highest dose of pitavastatin in patients at high risk of developing diabetes during a 3-year follow-up.

**Methods:**

This post hoc analysis of a prospective, single-blinded, randomized study compared the risk of NOD between the highest dose of pitavastatin (4 mg) and the lowest dose of pitavastatin (1 mg) over a 3-year follow-up in patients with acute coronary syndrome. Among 1044 patients of the original study, 667 patients at high risk of developing type 2 diabetes mellitus were in the subgroup analysis. The primary endpoint was a comparison of the differences in the cumulative incidence of NOD in the pitavastatin 1 mg and 4 mg groups during a 3-year follow-up.

**Results:**

With propensity score matching, there were no significant differences in baseline demographic characteristics between the 2 groups. Incidence of NOD was similar between the pitavastatin 1 mg and 4 mg groups [12 of 289 patients (4.2%) and 8 of 289 patients (2.8%), respectively; p = 0.36]. In a prespecified analysis, there were no significant differences in NOD events according to sex, age, diagnosis, body mass index, glucose intolerance, or dyslipidemia.

**Conclusions:**

Administration of highest-dose pitavastatin did not increase the risk of NOD in patients at high risk of developing diabetes during the 3-year follow-up. Moreover, various risk factors for NOD such as metabolic syndrome components, glucose intolerance, dyslipidemia, obesity, or hypertension did not affect the development of NOD during pitavastatin administration. Thus, the highest dose pitavastatin can be safely used in patients with metabolic syndrome who are at high risk of developing diabetes.

*Trial registration* Clinical Trial registration information. URL: https://clinicaltrials.gov/ct2/show/NCT02545231. Unique identifier: NCT02545231

## Background

Statins are well known to reduce the risk of cardiovascular events and improve clinical outcomes across a broad spectrum of patients [1, 2]. Moreover, higher dose statin therapy more significantly reduces cardiovascular events in high-risk patients [3]. Previous studies, however, have suggested a relationship between statin therapy and new-onset diabetes (NOD) [4–7]. A meta-analysis has shown that statin therapy increased the risk of NOD by 9% and that intensive therapy additionally increased the risk of NOD by 12% [6, 8].

Interestingly, the risk of NOD seems to vary according to the type and dose of statins. Previous studies reported that various types of statins, including atorvastatin, rosuvastatin, and simvastatin, increased the risk of NOD [6, 8, 9]. Higher doses of statin therapy further increased the hazard ratio of NOD [10]. However, high-dose pitavastatin is not considered to be associated with NOD [11]. Pitavastatin 2 mg showed a similar effectiveness in improving lipid profiles to that of atorvastatin 10 mg in Asian patients, with similar safety parameters [12]. When compared to other statins, the beneficial effects of pitavastatin on glucose metabolism or NOD may stem from the fact that it does not impair the differentiation and maturation of 3T3-L1 preadipocytes and does not suppress glucose transporter type 4 (GLUT4) expression [13].

Known risk factors for NOD during statin therapy are fasting blood glucose (FBG) ≥ 100 mg/dL, fasting triglycerides ≥ 150 mg/dL, body mass index (BMI) ≥ 30 kg/m^2^, and a history of hypertension (HTN) [14]. In the Justification for Use of statins in Prevention: an Intervention Trial Evaluating Rosuvastatin (JUPITER) trial, the risk factors for developing diabetes mellitus (DM) included at least 1 of the following: metabolic syndrome, impaired fasting glucose, BMI ≥ 30 kg/m^2^, or HbA1c > 6% [15]. Dyslipidemia can be controlled by the administration of relatively lower dose statins in Asian patients [16]. However, there are limited data regarding the impact of chronic pitavastatin use on the development of NOD in the Asian population [17, 18]. We prospectively compared the differences in the development of NOD between the lowest dose of pitavastatin and the highest dose of pitavastatin in patients at high risk of developing DM during a 3-year follow-up.

## Methods

### Study patients

Patients aged 30 to 79 years were eligible for the original trial of this study if they were (1) diagnosed with acute coronary syndrome (ACS) that was successfully treated with coronary stent implantation (TIMI flow grade 3 after the procedure). A total of 2463 consecutive patients were screened for inclusion at the Korea University Anam Hospital Cardiovascular Center between March 2013 and April 2015. Exclusion criteria were: (1) hypersensitivity to pitavastatin; (2) serum creatinine > 2.0 mg/dL; (3) hemoglobin A_1c_ > 9%; (4) type 1 DM; (5) serum platelet level < 100,000/μL; (6) left main coronary artery lesion; (7) left ventricular ejection fraction < 40%; (8) hepatic dysfunction (aspartate aminotransferase or alanine aminotransferase > twice the upper limit); (9) gastrointestinal disorders, such as Crohn’s disease; (10) alcohol abuse; (11) steroid or hormone replacement therapy; (12) life expectancy less than 1 year; (13) those with a known pregnancy, breast feeding, or having an intention to become pregnant during the study period; (14) any condition that would make participation in this study unsafe or unsuitable in the opinion of the investigator or (15) a lack of follow-up data.

**Fig. 1:**
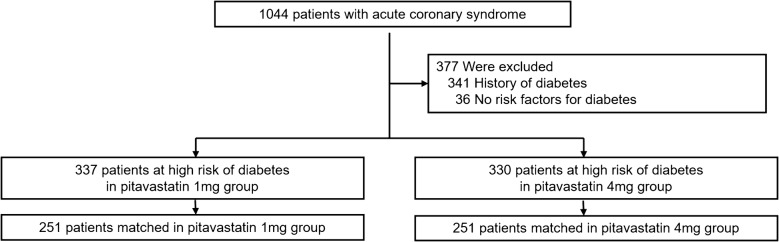
Study protocol. A total of 1044 patients underwent randomization in a 1:1 ratio to receive lowest dose (1 mg) or highest dose pitavastatin (4 mg) therapy for 3 years. After propensity score matching, 289 patients were enrolled in each group

Among these patients, we enrolled the patients who fulfilled at least 1 of following criteria for a high risk of developing type 2 DM: FBG ≥ 100 mg/dL, fasting triglycerides ≥ 150 mg/dL, BMI ≥ 25 kg/m^2^ according to the Korean guidelines of obesity, or presence of HTN (Fig. 1) [19]. To investigate the impacts on NOD, we excluded the patients with an existing diagnosis of DM.

### Study design

This study was a post hoc analysis of the prospective, open-label, single-blinded, randomized trial. A total of 1044 patients received lowest-dose pitavastatin (1 mg) or highest-dose pitavastatin (4 mg) for 3 years in the original study. Among them, 667 patients were at high risk of developing type 2 DM in the subanalysis (Fig. 1). Patients received randomization numbers sequentially from a secret randomization list that was computer generated in blocks of 3 by individuals who had no contact with the persons who assigned the patients to study groups or performed any of the assessments. Participants were unaware of the randomization assignments until the final data were obtained. The study was approved by the Korea University Hospital Institutional Review Board, and written informed consent was obtained from all participants or their legal guardians before their inclusion in the study. All clinical investigations were conducted according to the principles of the Declaration of Helsinki.

### Endpoints

The primary endpoint was a comparison of the differences in the cumulative incidence of NOD in the pitavastatin 1 mg and pitavastatin 4 mg groups during a 3-year follow-up. NOD was defined as ≥ HbA1c 6.5% or the current use of hypoglycemic agents according to the physician’s discretion. The secondary endpoints were the predictors of NOD and comparison of the changes in vascular function. Vascular function was evaluated by brachial-ankle pulse wave velocity (PWV), central blood pressure (BP), and augmentation index (AI). A patient was defined as an alcoholic when an average of ≥ 7 units of alcohol among men and ≥ 5 units among women were consumed for 2 days per week.

### Pulse wave velocity

All patients were evaluated for PWV at baseline and at the 36-month follow-up. After 5 min of rest in the supine position, PWV was measured using a volume plethysmographic apparatus (BP-203 RPE II; Colin, Komaki, Japan), which simultaneously recorded the PWV and the brachial and ankle BP on the left and right sides.

### Measurements of central BP and AI

Central BP recordings were obtained using the Omron HEM-9000AI cSBP device (Omron Healthcare, Kyoto, Japan) according to the manufacturer’s and user’s manuals. BP measurement was obtained via the digital oscillometric method using a BP cuff. The accompanying AI calculation was based on the patient’s pressure waveforms calibrated using the brachial systolic and diastolic BP. AI was determined by the change in pressure between the first and second peaks divided by the pulse pressure (AI = ΔP/PP). The first peak was obtained when the blood was ejected from the aorta. The second pressure peak occurred when the blood reflected at the aortic bifurcation. The pulse pressure was the overall peak pressure. All data were stored and analyzed off-line after the completion of testing.

### Laboratory analysis

Venous blood samples were drawn from each patient after fasting for 8 h or overnight. Blood samples were centrifuged to obtain plasma that was stored at − 80 °C. Plasma glucose was measured using the glucose oxidase method, and serum insulin levels were measured using an immunoradiometric assay (Biosource, Nivelles, Belgium). Total cholesterol, triglyceride, high-density lipoprotein (HDL) cholesterol, and low-density lipoprotein (LDL) cholesterol levels were determined using enzymatic methods with standard biochemical procedures on a BM Hitachi automated clinical chemistry analyzer (Hitachi, Tokyo, Japan).

### Statistical analysis

Data were expressed as mean ± standard deviation for the continuous variables, and as number and percentage of patients for the categorical variables. Fisher’s exact test or Chi-square test was used for categorical variables. The change from baseline was calculated as the value obtained at the end of treatment subtracted from the pre-treatment value. The results of the 2 groups were compared using the unpaired Student’s *t* test, and the comparisons of the results obtained before and after the treatment were analyzed using the paired *t* test. To balance the distribution of baseline characteristics, we used propensity score matching. We estimated a propensity score for each study participant using the multivariable logistic regression model. In the model, potential confounders and variables, such as age, sex, alcohol intake and smoking status, BMI, HTN, DM, and medication history were included. We then created an exchangeable comparison group of patients receiving pitavastatin 1 mg by matching each with a patient in the pitavastatin 4 mg group. The model was fit to the data during all steps of the regression analyses (Hosmer and Lemeshow goodness-of-fit test χ^2^ = 6.30, *p *= 0.85, and relative multivariate imbalance L1 after matching = 0.96). Using the propensity scores, we matched 251 of those patients receiving pitavastatin 1 mg to another 251 patients receiving pitavastatin 4 mg who had a similar propensity score. Our assessment of the covariate balance after matching focused on these standardized differences. p value < 0.05 was considered statistically significant. SAS software (version 9.3; SAS Institute, Cary, NC, USA) was used for the statistical analyses.

## Results

### Patient characteristics

Baseline patient characteristics between the pitavastatin 1 mg (n = 337) and pitavastatin 4 mg (n = 330) groups were significantly different. Mean age, body mass index, medications on admission, and lipid profiles showed statistically significant differences between the 2 groups (Table 1). After propensity score matching to balance the distribution of the baseline characteristics, there were no significant differences in baseline demographic characteristics between the 2 groups (Table 2). Follow-up was done 929.4 ± 312.5 and 940.2 ± 293.0 days after the index procedure in the pitavastatin 1 mg and pitavastatin 4 mg groups, respectively (p = 0.70).

**Table 1:** Baseline demographic and angiographic characteristics

Variable	Pitavastatin 1 mg (n = 337)	Pitavastatin 4 mg (n = 330)	p-value
Age (years)	64.1 ± 11.9	62.2 ± 11.8	0.04
Male sex	230 (68.2)	253 (76.7)	0.02
Body mass index (kg/m^2^)	24.3 ± 3.0	24.8 ± 3.1	0.03
Diagnosis			0.99
Unstable angina	210 (72.7)	209 (72.3)	
Non-ST elevation MI	45 (15.6)	45 (15.6)	
ST elevation MI	34 (11.8)	35 (12.1)	
Risk factors			
Hypertension	194 (57.6%)	183 (55.5%)	0.59
Smoking status			0.01
Current smoker	66 (19.6%)	98 (29.7%)	
Ex-smoker	64 (19.0%)	61 (18.5%)	
Never smoker	207 (61.4%)	171 (51.8%)	
Alcoholics	54 (16.0%)	63 (19.1%)	0.31
Prior MI	19 (5.4%)	13 (3.8%)	0.29
Medication on admission			
Aspirin	335 (99.4%)	330 (100%)	0.50
Clopidogrel	295 (87.5%)	263 (79.7%)	0.01
Ticagrelor	49 (14.5%)	63 (19.1%)	0.12
Prasugrel	10 (3.0%)	29 (8.8%)	0.001
ACE inhibitor	69 (20.5%)	60 (18.2%)	0.49
ARB	114 (33.8%)	94 (28.5%)	0.16
β-blocker	142 (42.1%)	158 (47.9%)	0.14
CCB	110 (32.6%)	116 (35.2%)	0.51
Diuretics	61 (18.1%)	54 (16.4%)	0.61
Laboratory findings			
Total cholesterol (mg/dL)	174.2 ± 41.7	185.2 ± 49.7	< 0.01
Triglyceride (mg/dL)	144.0 ± 105.9	164.5 ± 175.0	0.08
HDL cholesterol (mg/dL)	43.8 ± 11.2	42.9 ± 9.9	0.28
LDL cholesterol (mg/dL)	119.6 ± 34.5	127.5 ± 37.1	0.01
Creatinine (mg/dL)	1.1 ± 1.5	0.9 ± 0.2	0.06
Uric acid (mg/dL)	5.5 ± 1.5	5.8 ± 1.6	0.09
hsCRP (mg/L)	5.9 ± 12.6	7.4 ± 17.5	0.26
Fasting glucose (mmol/L)	113.6 ± 27.8	116.4 ± 28.4	0.28
HbA_1c_ (%)	5.8 ± 0.4	5.8 ± 0.3	0.89

Values are presented as mean ± standard deviation or n (%)

*ACE* angiotensin converting enzyme, *ARB* angiotensin receptor blocker, *CCB* calcium channel blocker, *HbA*_*1c*_ hemoglobin A_1c_, *HDL* high-density lipoprotein, *hsCRP* high-sensitivity C-reactive protein, *LDL* low-density lipoprotein, *MI* myocardial infarction

**Table 2:** Baseline demographic characteristics after propensity score matching

Variable	Pitavastatin 1 mg (n = 251)	Pitavastatin 4 mg (n = 251)	p-value
Age (years)	63.3 ± 12.2	62.6 ± 11.9	0.54
Male sex	177 (70.5%)	185 (73.7%)	0.49
Body mass index (kg/m^2^)	24.4 ± 3.0	24.6 ± 3.0	0.66
Diagnosis			0.88
Unstable angina	184 (73.3%)	181 (72.1%)	
Non-ST elevation MI	37 (14.7%)	41 (16.3%)	
ST elevation MI	30 (12.0%)	29 (11.6%)	
Risk factors			
Hypertension	142 (56.6%)	144 (57.4%)	0.93
Smoking status			0.94
Current smoker	64 (25.5%)	65 (25.9%)	
Ex-smoker	46 (18.3%)	43 (17.1%)	
Never smoker	141 (56.2%)	143 (57.0%)	
Alcoholics	46 (18.3%)	44 (17.5%)	0.91
Prior stroke	0 (0.0%)	0 (0.0%)	–
Medication on admission			
Aspirin	251 (100.0%)	251 (100.0%)	–
Clopidogrel	215 (85.7%)	204 (81.3%)	0.23
Ticagrelor	40 (15.9%)	48 (19.1%)	0.41
Prasugrel	10 (4.0%)	20 (8.0%)	0.09
ACE inhibitor	41 (16.3%)	49 (19.5%)	0.42
ARB	82 (32.7%)	78 (31.1%)	0.77
β-blocker	117 (46.6%)	118 (47.0%)	0.99
CCB	86 (34.3%)	84 (33.5%)	0.93
Diuretics	49 (19.5%)	44 (17.5%)	0.65
Laboratory findings			
Total cholesterol (mg/dL)	177.9 ± 41.4	180.8 ± 46.6	0.46
Triglyceride (mg/dL)	145.7 ± 109.7	162.6 ± 182.6	0.21
HDL cholesterol (mg/dL)	43.4 ± 10.6	42.9 ± 9.9	0.60
LDL cholesterol (mg/dL)	122.9 ± 34.2	123.4 ± 34.2	0.88
Creatinine (mg/dL)	1.1 ± 1.5	0.9 ± 0.2	0.08
Uric acid (mg/dL)	5.6 ± 1.5	5.8 ± 1.6	0.23
hsCRP (mg/L)	5.6 ± 11.8	6.7 ± 15.9	0.43
Fasting glucose (mmol/L)	115.4 ± 29.3	114.9 ± 26.3	0.85
HbA_1c_ (%)	5.8 ± 0.4	5.8 ± 0.3	0.36

Values are presented as mean ± standard deviation or n (%)

*ACE* angiotensin converting enzyme, *ARB* angiotensin receptor blocker, *CCB* calcium channel blocker, *HbA*_*1c*_ hemoglobin A_1c_, *HDL* high-density lipoprotein, *hsCRP* high sensitivity C-reactive protein, *LDL* low-density lipoprotein, *MI* myocardial infarction

### NOD and clinical outcomes during the 3-year follow-up

**Fig. 2:**
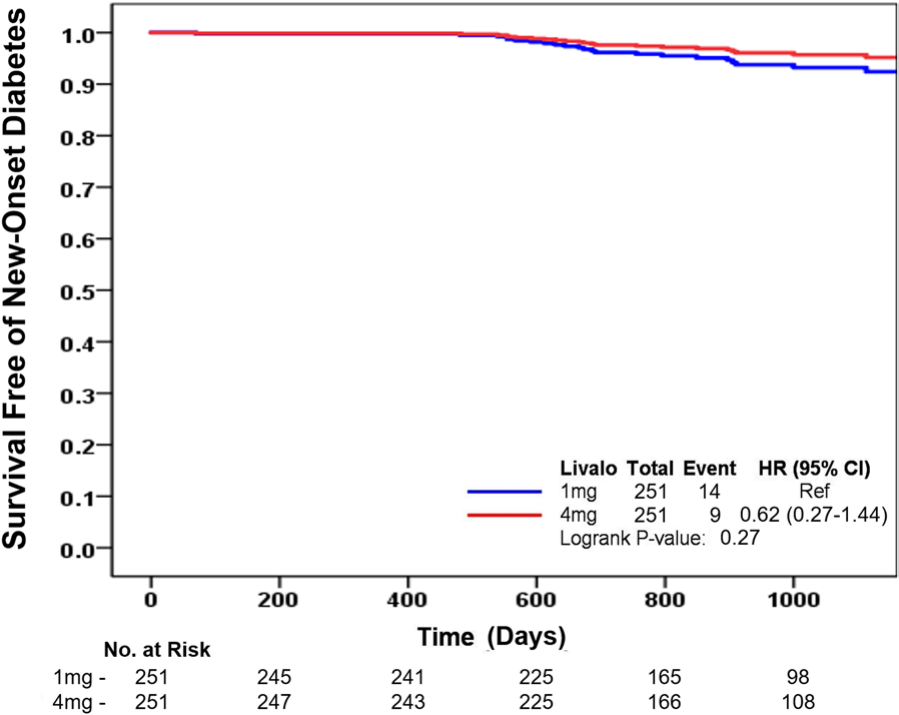
Survival without new-onset diabetes in the pitavastatin 1 mg and pitavastatin 4 mg groups during the 3-year follow-up

The incidence of NOD was similar between the pitavastatin 1 mg and the pitavastatin 4 mg groups during the 3-year follow-up [14 of 251 patients (5.6%) and 9 of 251 patients (3.6%), respectively; p = 0.39] (Table 3 and Fig. 2). In a prespecified analysis, there were no significant differences in NOD events that occurred at less than 1 year or more than 1 year after baseline randomization between the pitavastatin 1 mg and the pitavastatin 4 mg groups [1 (0.4%) and 0 (0.0%), p = 0.99 during the first year of follow-up and 13 (5.2%) and 9 (3.6%), p = 0.39 more than 1 year after baseline randomization, respectively]. In the analyses of the separate clinical events, the incidences of each event were similar between the 2 groups.

**Table 3:** Incidence of new-onset diabetes and clinical events during the 3-year follow-up

Variable	Pitavastatin 1 mg (n = 251)	Pitavastatin 4 mg (n = 251)	p-value
New-onset diabetes	14 (5.6%)	9 (3.6%)	0.39
Baseline ~ 1 year	1 (0.4%)	0 (0.0%)	0.99
> 1 year	13 (5.2%)	9 (3.6%)	0.39
All cause death	9 (3.6%)	8 (3.2%)	0.99
Cardiac death	3 (1.2%)	1 (0.4%)	0.62
Non-fatal myocardial infarction	14 (5.6%)	8 (3.2%)	0.28
Ischemic stroke	5 (2.0%)	2 (0.8%)	0.45
Target lesion revascularization	12 (4.8%)	9 (3.6%)	0.66
Target vessel revascularization	18 (7.2%)	13 (5.2%)	0.46
Non-target lesion/vessel revascularization	7 (2.8%)	15 (6.0%)	0.13

Values are presented as n (%)

### Predictors of NOD

**Fig. 3:**
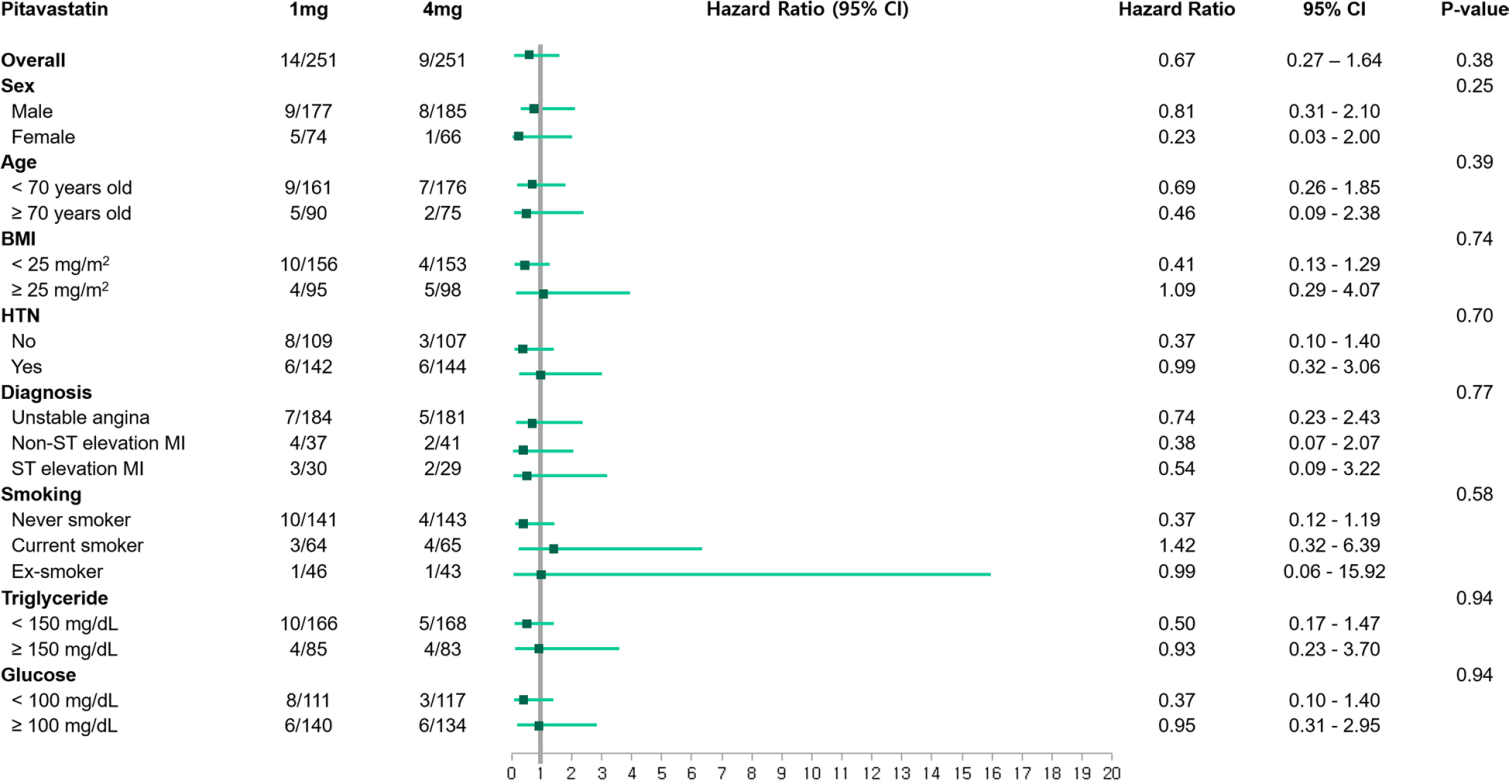
Incidences of new-onset diabetes in selected, prespecified subgroups

The number of metabolic syndrome components did not affect the occurrence of NOD (Table 4). Total occurrences of NOD did not increase along with the increased number of metabolic syndrome components. Moreover, administration of pitavastatin 4 mg did not increase the risk of NOD compared to pitavastatin 1 mg. Incidences of NOD according to sex, age, BMI, level of TG and glucose, HTN, initial diagnosis at randomization, and smoking status were comparable between the 2 groups (Fig. 3). In the univariate analysis with the propensity score-matched population, pitavastatin 4 mg did not significantly increase the risk of NOD (Table 5). The number of metabolic syndrome components and other factors did not show significant differences in terms of the development of NOD.

**Table 4:** Incidence of new-onset diabetes according to the number of metabolic syndrome components during the 3-year follow-up

Number of metabolic syndrome components	Incidence of new-onset diabetes		HR (95% CI)	p-value
	Pitavastatin 1 mg	Pitavastatin 4 mg		
1	4/50	1/51	0.23 (0.03, 2.13)	0.20
2	5/96	4/100	0.75 (0.20, 2.91)	0.69
3	4/73	2/74	0.48 (0.09, 2.70)	0.40
4	1/32	2/26	2.58 (0.22, 30.20)	0.45
Overall	14/251	9/251	0.67 (0.27, 1.64)	0.38

**Table 5:** Predictors for new-onset diabetes

Risk factor	Univariate analysis			
	HR	95% CI		p-value
		Lower	Upper	
Pitavastatin 4 mg	0.63	0.27	1.48	0.29
Number of metabolic syndrome components				
1	–			
2	0.92	0.30	2.83	0.89
3	0.82	0.24	2.75	0.74
4	1.05	0.24	4.55	0.95
Age	0.99	0.65	1.03	0.63
Female	0.91	0.35	2.35	0.84
Body mass index	1.01	0.88	1.16	0.91
Hypertension	0.82	0.35	1.89	0.64
Total cholesterol	1.01	0.99	1.01	0.86
Triglyceride	1.00	0.99	1.01	0.82
HDL-cholesterol	0.96	0.92	1.01	0.11
LDL-cholesterol	1.00	0.99	1.02	0.58
hsCRP	1.01	0.99	1.04	0.23
Fasting glucose	1.01	0.99	1.02	0.15
HbA1c	6.61	0.69	63.18	0.10

*HbA*_*1c*_ hemoglobin A_1c_, *HDL* high-density lipoprotein, *hsCRP* high sensitivity C-reactive protein, *LDL* low-density lipoprotein

### Changes in inflammatory markers, lipid profiles, and vascular function during the 3-year follow-up

Decreases in LDL cholesterol levels from baseline were significantly greater in the pitavastatin 4 mg group than in the pitavastatin 1 mg group during the 3-year follow-up (− 37.5 ± 37.6 mg/dL and − 15.2 ± 39.3 mg/dL, respectively; p < 0.05) (Additional file 1: Table S1). The percent reduction of LDL cholesterol from baseline was 12.0% in the pitavastatin 1 mg group and 31.0% in the pitavastatin 4 mg group. No significant differences in the PWV, ankle-brachial index, central BP, or AI were detected between the groups (Additional file 1: Table S2).

## Discussion

This post hoc analysis of a prospective, single-blinded, randomized study compared the risk of NOD between the highest dose of pitavastatin (4 mg) and the lowest dose of pitavastatin (1 mg) over a 3-year follow-up in patients with ACS. Administration of pitavastatin, including the highest-dose pitavastatin (4 mg), did not increase the risk of NOD during the 3-year follow-up; moreover, this study demonstrated that the patients with metabolic syndrome who were at high risk of NOD could safely receive the highest-dose pitavastatin without further increasing the risk of NOD.

### Statin therapy and NOD risk

The JUPITER trial revealed that the use of rosuvastatin significantly increased the risk of NOD [20]. In a meta-analysis of randomized controlled trials, the administration of statins increased the risk of NOD by 10–13% [6]. More importantly, higher doses of statins considerably amplified the NOD risk [8]. Despite this less good glycemic control and NOD risk, various guidelines have recommended the use of statin therapy to reduce future major adverse cardiovascular events (MACEs), because the beneficial effects of statin were significantly greater than the known side effects of statins, especially in ACS patients [21]. However, individual assessment to minimize the risk of NOD when administering statins is needed. Although epicardial adipose tissue thickness at systole can be a useful marker for predicting NOD during high‑intensity statin therapy in a previous study, there are inconsistent findings with the pitvastatin [22].

### Mechanisms of pitavastatin

Previous studies have suggested several mechanisms of the occurrence of NOD after statin therapy [23]. Although the strong LDL cholesterol lowering effects of statins are driven by the inhibition of HMG-CoA reductase, the isoprenoid levels are down-regulated during this process. Consequently, glucose uptake through GLUT4 in adipocytes may be decreased. In addition, statins directly block glucose-induced calcium channels in pancreatic β-cells and reduce insulin signal transduction. Unlike other statins, pitavastatin has shown at least neutral effects on the development of NOD. It was reported that pitavastatin significantly increased adiponectin levels, which may contribute to its anti-inflammatory and anti-diabetic properties [24]. A pharmacokinetic study of pitavastatin revealed higher systemic bioavailability, leading to increased extrahepatic effects on adipose tissue and circulating adiponectin levels [25]. In previous in vivo studies, adiponectin-deficient mice showed insulin-resistant and glucose-intolerant characteristics and supplementation of adiponectin improved glucose metabolism [26]. It has been well known that the expression of adiponectin receptors is decreased in obese patients, which is associated with the development of DM and atherosclerosis [27]. In addition, significant increases in HDL cholesterol level by pitavastatin could beneficially influence glucose metabolism by facilitating glucose uptake and enhanced insulin sensitivity [28]. Pitavastatin may also preserve adipocyte maturation and glucose transporter GLUT4 expression, which could be decreased with the use of other statins [29].

### Pitavastatin and NOD risk

Recently, a study reported that the administration of higher dose pitavastatin significantly decreased the incidence of future MACEs without increasing the risk of NOD compared to a lower dose of pitavastatin [30]. In that study, the overall incidence rate of NOD with pitavastatin was 4.3%. However, the risk of NOD may escalate according to patients’ baseline levels of FBG and triglycerides or BMI. In our study, we enrolled patients who were at high risk of developing NOD and specifically compared the development of NOD according to the number of risk factors for DM. We found that a higher dose of pitavastatin did not increase the risk of NOD. In addition, the number of metabolic syndrome components did not affect the risk of NOD with pitavastatin administration. Although previous studies have reported that the NOD risk was significantly elevated in older patients and women, there were no significant differences in the NOD incidences among those groups in our study [6, 31]. Moreover, any spike in NOD incidence within 1 year after the administration of statins should be carefully evaluated to assess whether it can be causally linked to statin exposure [10]. In our study, comparable incidences of NOD before and after 1 year were observed.

In a previous study, while the administration of statins definitely reduced the incidences of cardiovascular events, pitavastatin was found to increase the NOD risk or worsen the hyperglycemia [32, 33]. However, the baseline characteristics of the retrospectively enrolled patients were heterogeneous and the number of cases was limited to accurately assess the effects on NOD. According to other Asian data, higher dose of pitavastatin improved the FBG level in contrast to other statins [34]. There were no ethnic differences between the efficacy or safety of pitavastatin in Asian and European [35]. Moreover, a meta-analysis demonstrated that pitavastatin did not elevate the FBG and HbA1c levels or the incidence of DM compared to placebo or to other statins [36]. Another study reported that early pitavastatin therapy in patients with metabolic syndrome could be safely administered without any deterioration in glucose intolerance [37]. These results were meaningful in that highest dose pitavastatin could be safely administered in patients at high risk of developing DM, such as those with metabolic syndrome.

The present study has a few limitations. This study was originally designed to investigate the differences in the occurrence of cardiovascular events between pitavastatin 1 mg and 4 mg groups. Moreover, since this study was confined to patients at high risk of developing type 2 DM, our findings should not be extrapolated to a broad spectrum of patients.

## Conclusions

In conclusion, when compared to the lowest dose of pitavastatin (1 mg), administration of the highest dose of pitavastatin (4 mg) did not increase the risk of NOD during the 3-year follow-up. Various risk factors for NOD, such as the number of metabolic syndrome components, glucose intolerance, dyslipidemia, obesity, and HTN, did not affect the development of NOD during pitavastatin therapy. Thus, patients with metabolic syndrome who are at high risk of NOD can safely receive the highest dose pitavastatin without increasing their risk of NOD.

## Supplementary information

**Additional file 1: Table S1.** Comparison of the changes in laboratory findings during the 3-year follow-up between the pitavastatin 1 mg and 4 mg groups. **Table S2.** Comparison of the changes in vascular function during the 3-year follow-up between the pitavastatin 1 mg and 4 mg groups.

## Data Availability

The datasets used during the current study are available from the corresponding author on reasonable request.

## Abbreviations

ACS: acute coronary syndrome
AI: augmentation index
BMI: body mass index
BP: blood pressure
DM: diabetes mellitus
FBG: fasting blood glucose
HTN: hypertension
JUPITER: Justification for Use of statins in Prevention: an Intervention Trial Evaluating Rosuvastatin trial
NOD: new-onset diabetes
PWV: pulse wave velocity
