# Supplementary material for: Incidence of new-onset diabetes with 1 mg versus 4 mg pitavastatin in patients at high risk of developing diabetes during a 3-year follow-up
Source: Cardiovasc Diabetol. 2019 Nov 21;18:162. doi: 10.1186/s12933-019-0969-z (PMC6868797; doi:10.1186/s12933-019-0969-z)
Supplement: Supplementary file 1 — Additional file 1: Table S1. Comparison of the changes in laboratory findings during the 3-year follow-up between the pitavastatin 1 mg and 4 mg groups. Table S2. Comparison of the changes in vascular function during the 3-year follow-up between the pitavastatin 1 mg and 4 mg groups. [file 12933_2019_969_MOESM1_ESM.docx]

**Table S1.** Comparison of the changes in laboratory findings during the 3-year follow-up between the pitavastatin 1 mg and 4 mg groups

| Variables | Pitavastatin 1 mg  (n=251) | | Pitavastatin 4 mg  (n=251) | |
| --- | --- | --- | --- | --- |
|  | Baseline | After 3 years | Baseline | After 3 years |
|  | Changes from baseline | | Changes from baseline | |
| Total cholesterol (mg/dL) | 177.9 ± 41.4 | 155.2 ± 31.7 | 180.8 ± 46.6 | 147.2 ± 32.6* |
|  | -23.8 ± 45.9 | | -33.9 ± 48.6* | |
| Triglyceride (mg/dL) | 145.7 ± 109.7 | 140.0 ± 66.7 | 162.6 ± 182.6 | 138.8 ± 101.6 |
|  | -2.3 ± 98.2 | | -32.5 ± 184.0* | |
| HDL-cholesterol (mg/dL) | 43.4 ± 10.6 | 47.3 ± 12.4 | 42.9 ± 9.9 | 45.5 ± 10.8 |
|  | 2.4 ± 10.0 | | 2.8 ± 9.3 | |
| LDL-cholesterol (mg/dL) | 122.9 ± 34.2 | 108.1 ± 31.3 | 123.4 ± 34.2 | 85.2 ± 24.3† |
|  | -15.2 ± 39.3 | | -37.5 ± 37.6† | |
| hsCRP (mg/L) | 5.6 ± 11.8 | 5.0 ± 14.4 | 6.7 ± 15.9 | 7.0 ± 26.7 |
|  | -0.7 ± 16.0 | | 1.4 ± 32.6 | |
| Fasting glucose (mmol/L) | 115.4 ± 29.3 | 114.6 ± 30.3 | 114.9 ± 26.3 | 109.7 ± 22.0 |
|  | -1.4 ± 31.9 | | -5.8 ± 36.0 | |
| HbA_1c_ (%) | 5.8 ± 0.4 | 6.2 ± 1.0 | 5.8 ± 0.3 | 5.9 ± 0.6* |
|  | -0.4 ± 1.0 | | 0.0 ± 0.4 | |

Values are presented as mean ± standard deviation

* p < 0.05 compared between pitavastatin 1mg and 4mg.

† p < 0.001 compared between pitavastatin 1mg and 4mg.

HbA_1c_ = hemoglobin A_1c_

HDL = high-density lipoprotein

hsCRP = high-sensitivity C-reactive protein

LDL = low-density lipoprotein

**Table S2.** Comparison of the changes in vascular function during the 3-year follow-up between the pitavastatin 1 mg and 4 mg groups

| Variables | Pitavastatin 1 mg  (n=251) | | Pitavastatin 4 mg  (n=251) | |
| --- | --- | --- | --- | --- |
|  | Baseline | After 3 years | Baseline | After 3 years |
|  | Changes from baseline | | Changes from baseline | |
| Right PWV, cm/s | 1672 ± 383 | 1601 ± 377 | 1624 ± 327 | 1576 ± 376 |
| Changes from baseline | -57 ± 267 | | -64 ± 358 | |
| Left PWV, cm/s | 1672 ± 359 | 1610 ± 378 | 1608 ± 324 | 1557 ± 350 |
| Changes from baseline | -61 ± 283 | | -55 ± 243 | |
| Right ABI, % | 1.10 ± 0.12 | 1.11 ± 0.11 | 1.10 ± 0.10 | 1.10 ± 0.10 |
| Changes from baseline | 0.01 ± 0.13 | | 0.00 ± 0.12 | |
| Left ABI, % | 1.10 ± 0.11 | 1.10 ± 0.11 | 1.09 ± 0.13 | 1.07 ± 0.13 |
| Changes from baseline | 0.00 ± 0.10 | | -0.01 ± 0.17 | |
| SBP, mmHg | 129.6 ± 19.4 | 138.8 ± 105.4 | 131.6 ± 18.5 | 128.1 ± 18.3 |
| Changes from baseline | 10.6 ± 127.5 | | -5.3 ± 21.2 | |
| DBP, mmHg | 76.4 ± 15.0 | 77.5 ± 15.2 | 76.7 ± 14.0 | 76.3 ± 13.8 |
| Changes from baseline | -2.9 ± 16.7 | | -0.7 ± 17.3 | |
| cSBP, mmHg | 131.3 ± 24.3 | 130.9 ± 20.7 | 132.6 ± 22.1 | 132.0 ± 21.6 |
| Changes from baseline | -0.9 ± 26.4 | | -3.3 ± 22.6 | |
| AI, % | 78.4 ± 14.2 | 78.6 ± 15.7 | 77.3 ± 14.1 | 80.7 ± 16.5 |
| Changes from baseline | -0.6 ± 17.3 | | 3.4 ± 13.2 | |

Values are presented as mean ± standard deviation

ABI = ankle-brachial index

AI = augmentation index

cSBP = estimated central systolic blood pressure

DBP = diastolic blood pressure

PWV = pulse wave velocity

SBP = systolic blood pressure
